# Fibronectin promotes tumor progression through integrin αvβ3/PI3K/AKT/SOX2 signaling in non-small cell lung cancer

**DOI:** 10.1016/j.heliyon.2023.e20185

**Published:** 2023-09-14

**Authors:** Jin-Long Wu, Cheng-Feng Xu, Xu-Hui Yang, Ming-Song Wang

**Affiliations:** a Department of Thoracic Surgery, Shanghai Ninth People's Hospital, School of Medicine, Shanghai Jiaotong University, Shanghai City, 200011, China; b Department of Pharmacy, Shidong Hospital of Shanghai Yangpu District, Shanghai City, 200438, China

**Keywords:** Non-small cell lung cancer, Fibronectin, Integrin αvβ3, PI3K/AKT signal

## Abstract

The tumor microenvironment, especially the extracellular matrix (ECM), is strongly associated with tumor cell proliferation and metastasis. Numerous studies have provided evidence suggesting that fibronectin (FN) in ECM supports cancer cell escape and contributes to cell migration, resulting in distant cancer metastasis and poor outcomes in patients. In our study, it was demonstrated that FN expression was elevated in tumor tissues from highly malignant NSCLC patients, compared to those with low malignancy (p = 0.0076). Importantly, FN promoted proliferative phenotypes and strengthened tumorigenesis capabilities in NSCLC cells, including A549 and Lewis cells, leading to sustained tumor growth *in vivo*. Mechanistically, it was identified that FN facilitated the activation of the integrin αvβ3/PI3K/AKT signaling pathway, which subsequently upregulated tumor stemness through the downstream transcription factor SOX2. Blockade of integrin αvβ3 signal efficiently suppressed NSCLC proliferation and tumorigenesis both *in vitro* and *in vivo*. In conclusion, our study demonstrated that extracellular FN could facilitate NSCLC development through the integrin αvβ3/PI3K/AKT/SOX2 signaling pathway. Blockade of integrin αvβ3 could efficiently enhance the anticancer effects of chemotherapy, offering an innovative approach for clinical NSCLC therapy.

## Abbreviations

Fibronectin: (FN)
Non-small cell lung cancer: (NSCLC)
Extracellular matrix: (ECM)
Paclitaxe: (PTX)
Methotrexate: (MTX)
Cilengitide trifluoroacetate: (CT)
Cancer associated fibroblasts: (CAFs)

## 1 Introduction

Lung cancer is one of the most common cancers worldwide and is the leading cause of carcinoma-related deaths. In the United States, over 225,000 patients are newly diagnosed with lung carcinoma annually, resulting in more than 160,000 deaths each year [1]. NSCLC is the predominant type of lung cancer, encompassing squamous cell carcinoma and adenocarcinoma, which collectively account for approximately 85% of all lung cancers [2]. Traditionally, surgical resection and chemotherapy are the standard clinical therapeutic options for patients with NSCLC, especially for those in the early stages [3]. However, the 5-year survival rate is approximately 70% for patients in stage I, and only 25% of stage III patients survive after surgical resection [4,5]. Furthermore, chemotherapy-treated patients can develop multi-drug resistance, causing the failure of chemotherapy or targeted therapy, thereby resulting in poor outcomes for patients with NSCLC [6]. Thus, our study seeks to elucidate the underlying mechanism of NSCLC development and explore innovative approaches to improve outcomes in NSCLC therapy.

Tumor cell proliferation and metastasis are strongly associated with the tumor microenvironment, including the factors of hypoxia, mesenchymal cell sub-population, tumor associated immune cells and the extracellular matrix (ECM) [7]. ECM serves as a structural foundation for intercellular communication and serves as a barrier against cellular invasion and malignant progression in cancer [8]. Various cellular integrin dimers can bind to these extracellular elements, subsequently manipulating changes and intracellular signaling [9,10]. Moreover, alterations in the ECM, such as laminin, hyaluronan, and FN, are increasingly recognized to contribute to tumor pathogenesis and various malignant behaviors in solid tumors [11]. Increasing evidence has suggested that stromal cells, especially CAFs, play a crucial role in extracellular matrix remodeling, which affects the activation/inactivation of integrins pro-survival signaling pathways in tumor cells. Notably, integrins, particularly integrin αvβ3, have been identified as cell adhesion receptors during tumor growth and metastasis, indicating the potential role of CAFs/extracellular matrix/integrins axis in tumor development.

Fibronectin is a critically essential extracellular matrix protein that regulates cell-matrix interaction during fundamental events, such as cell development, wound healing, fibrosis, and tumor progression. Previous studies have demonstrated that FN appears to promote tumor cell invasion and migration by guiding and supporting tumor cells to escape from the primary site, leading to tumor invasion and distant metastasis [12]. Other reports have indicated that FN is also capable of directly stimulating tumor cell proliferation and suppressing apoptosis in ovarian cancer [13]. However, the origin of cellular FN in tumor microenvironment is still controversial, and the underlying mechanism of tumor cell proliferation induced by FN is still poorly characterized. Specifically, pro-tumor processes induced by FN have rarely been investigated in NSCLC. Innovative approaches to target FN-induced tumor progression might be an attractive strategy for clinical combination therapy in NSCLC. Given these challenges and the current research gap on the role of FN in NSCLC, this study aims to explore the role of FN in NSCLC development, investigate the underlying mechanism by which FN promotes tumor growth and provide innovative approaches for clinical combination therapy in NSCLC.

## 2 Materials and methods

### 2.1 Cell lines and reagents

We purchased human lung cancer cell line A549 and murine lung cancer cell line Lewis (https://web.expasy.org/cellosaurus/CVCL_S007) from the American Type Culture Collection (ATCC). A549 cells were cultured in RPMI-1640 complete medium (Gibco, MA, USA) supplemented with 10% fetal calf serum (Gibco, MA, USA), at 37 °C in 5% CO_2_ atmosphere (from 2015–6 to 2022–3). Lewis cells were cultured in DMEM complete medium (Gibco, MA, USA) supplemented with 10% fetal calf serum (Gibco, MA, USA) under the same conditions. Paclitaxel (PTX) and methotrexate (MTX) were purchased from Sangon Biotech (Shanghai, China). Integrin avβ3 inhibitor Cilengitide trifluoroacetate (CT), PI3K inhibitor LY294002, and AKT inhibitor MK-2206 were purchased from Selleck Chemicals (MA, USA), and FN was purchased from Sigma (MA, USA). Tumor cells were treated with inhibitors at a concentration of FN (100 ng/ml), CT (200 ng/ml), LY204002 (0.5 μM) or MK2206 (10 nM).

### 2.2 Tumor tissues screening and collection from NSCLC patients

Primary NSCLC tissues were sterilely obtained after the surgery at the Shanghai Ninth People's Hospital. Samples were divided into high malignant (stage T3, n = 10) and low malignant (stage T1, n = 10) groups according to clinical NSCLC stage (Supplementary Table 1). The study was approved by the Ethics Committee of Shanghai Ninth People's Hospital. All samples collection and processing were carried out adhered to the principles outlined in the Declaration of Helsinki. All patients signed informed consent prior to tumor tissues collection treatment, including allowing their data to be used for further research. All experiments were performed under the supervision of the Ethics Committee of Shanghai Ninth People's Hospital. To isolate human cancer associated fibroblasts (CAFs), immune cells and cancer cells from patients' tumor tissue, we cut tumor tissues into pieces as small as possible, then digested them with RPMI-1640 complete culture medium (Gibco, MA, USA) containing cell aggregate dissociation medium ACCUMAXTM (A7089, Sigma, MA, USA) at 37 °C, 5% CO_2_ incubator for 2 h. Next, the digested cells isolated from tumor tissues were washed with PBS and seeded into a 6-well plate with 2 ml of RPMI-1640 medium supplemented with 10% fetal bovine serum at 37 °C (1 × 10^6^ cells per well). After 2 h, the adherent macrophages were removed and those suspending cells were transferred to another 6-well plate. After 12 h, the suspending cells were collected to sort CD 45+ cells as immune cells and CD 90+ cells as CAFs. The adherent cells were cultured again. After 3 days, those adherent cells were collected to sort CD 45-and CD90^−^as cancer cells for further analysis.

### 2.3 Cell proliferation analysis and colony formation experiments

The cell proliferation was assessed by using Cell Counting Kit-8 (CCK-8, Solarbio, Beijing, China), Briefly, cancer cells were seeded into 96 well culture plate (2000 cells per well) and cultured at 37 °C, 5% CO_2_ incubator. After 72 h, 10 μl CCK-8 solution was added into the 96-well plates and incubated it at 37 °C for 90 min. The absorbance at 450 nm was measured by a microplate reader (Bio-Rad, Hercules, USA), each experiment was performed independently at least three times.

For colony formation analysis, a 3D soft fibrin gel system was employed to evaluate the colony formation capability in this study [14]. Specifically, 1000 cancer cells were seeded into the 3D soft gel (90 Pa) with RPMI-1640 complete culture medium or DMEM culture medium. After 3 days, the colony formation ratios were calculated. Each experiment was performed at least three times independently.

### 2.4 Flow cytometry

Cell precipitation was suspended and washed with PBS supplemented with 2%BSA, and then the cells were stained with the anti-CD90 primary antibody (Abcam, Cambridge, UK) and anti-CD45 primary antibody (Abcam, Cambridge, UK) for 30 min in 4 °C.The isotype was used as a negative control. Ultimately, the samples were sorted by DAKO Flow Cytometer (DAKO, CA, USA).

### 2.5 Real-time PCR

We detected the targeted gene expression through quantitative real-time PCR, and 1ug cDNA was used as template for amplification with SYBR Green Real-Time PCR master mixes (Thermo Fisher Scientific, MA, USA). We used GAPDH as the internal control and normalized the target gene level to the GAPDH by the ΔΔC_t_ method to quantify the relative expression. Notably, three independent experiments were performed in each sample. The primer pairs in our study were listed in supplementary information (Supplementary Table 2).

### 2.6 RNA interference

The A549 SOX2 silence was performed using siRNA technology. SiRNA transfection was conducted with the Lipofectamine RNAi-MAX kit (Thermo, MA, USA) in RPMI 1640 medium according to the manufacturer instruction. Briefly, Lewis or A549 cells were cultured in RPMI-1640 medium without FBS in 6-well plates for 24 h and reached 50% confluence. The cells were transfected with 100 nM siRNA by Lipofectamine RNAi-MAX kit in serum free culture medium for 6 h, and were incubated in normal RPMI-1640 medium for 72 h. Sequences of SOX2 siRNA were used of following sequence: siRNA #1 5‘-AAAACCAAGACGCTCATGAAG -3’ and siRNA #2 5‘-ACCTCCGGGACATGATCAGCA-3’, scramble RNA 5′-AACCTCCGGTATACCCTTATG-3’.

### 2.7 Western blotting

Cell pellets were lysed by adding RIPA Lysis Buffer (Beyotime, Beijing, China), and an equal amount of protein was boiled with loading buffer for 7min. Then, protein was separated by SDS–PAGE, and transferred onto polyvinylidene difluoride (PVDF). The PVDF membrane was blocked with 5% BSA and incubated overnight at 4 °C with mouse monoclonal anti-integrin αvβ3 (1:500, ab7166, Abcam, UK), rabbit monoclonal p-PI3K (1:500, ab278545, Abcam, Cambridge, UK), rabbit monoclonal total-PI3K (1:500, ab302958, Abcam, Cambridge, UK), rabbit polyclonal p-AKT (1:500, ab38449, Abcam, Cambridge, UK), rabbit polyclonal total-AKT (1:500, Abcam, ab8805, Cambridge, UK) and mouse monoclonal actin (1:500, ab8826, Abcam, Cambridge, UK). Next day, the sample was incubated with horse radish peroxidase (HRP) conjugated secondary antibody (1:1000, Abcam, Cambridge, UK) for 1 h at room temperature and then visualized by the chemiluminescence (ECL) Detection Kit (CST, Boston, MA, USA). In this study, all uncropped blotting figures were deposited in supplementary materials.

### 2.8 Immunofluorescence staining

The pathological sections of NSCLC patients’ tumor tissues were retrieved by microwave antigen retrieval (Citrate-EDTA Antigen Retrieval Solution, Beyotime). The sections were then blocked with 5% BSA and incubated with mouse monoclonal anti-integrin αvβ3 (1:100, ab7166, Abcam, UK), rabbit monoclonal SOX2 (1:200, ab92494, Abcam, Cambridge, UK), rabbit polyclonal p-AKT (1:200, ab38449, Abcam, Cambridge, UK), rabbit monoclonal KLF (1:200, ab215036, Abcam, Cambridge, UK), rabbit monoclonal total-PI3K (1:500, ab302958, Abcam, Cambridge, UK), mouse monoclonal CD90 (1:100, ab181496, Abcam, Cambridge, UK) for 4 °C overnight, and followed by horse radish peroxidase (HRP) conjugated secondary antibodies (1:600; Abcam, Cambridge, UK), and the nucleus was stained with DAPI. In addition, the A594 and Lewis cells were fixed in 4% paraformaldehyde, permeabilized with 0.2% triton-X100, and blocked by 5%BSA, followed by incubating with anti-SOX2 antibody (1:200, Abcam, Cambridge, UK) for 4 °C overnight, and followed by HRP conjugated secondary antibodies (1:600; Abcam, Cambridge, UK), and the nucleus was stained with DAPI. All immunofluorescence images were captured from FV1000 laser scanning confocal microscope (Leica, Barnack, Germany) and analyzed by IPP software.

### 2.9 Immunohistochemistry

The pathological sections of NSCLC patients’ tumor tissues were retrieved by microwave antigen retrieval (Citrate-EDTA Antigen Retrieval Solution, Beyotime, Beijing, China). The sections were blocked with 5% BSA and incubated with rabbit polyclonal FN antibody (1:200, ab2431, Abcam, Cambridge, UK) and mouse monoclonal CD90 (1:100, ab181496, Abcam, Cambridge, UK) overnight at 4 °C. Signal amplification staining was performed using the ABC HRP Kit (Thermo, MA, USA) and counterstained with hematoxylin. Next, samples were treated with dehydration, cleaned with xylene and covered with neutral resin. Sections were then captured with microscope (Leica, Barnack, Germany). To further analyze the number of CD90 positive CAFs in tumor tissues, we randomly selected 25 views of the IHC sections. The number of CD90 positive cells were counted, and the average of 25 views were calculated. The low malignant group were set as control (value = 1), and the high malignant group were calculated relative to the control (Fig. S1A). The relative expression of FN was analyzed by using the Image-Pro Plus 2.0 software.

### 2.10 Animal protocol

6∼8 weeks female C57BL mice and nude mice (BalB/C) used in this study were purchased from Huafukang Company (Beijing, China) and housed in the SPF facility. An orthotopic NSCLC model was established by instilling A549 (1 × 10^6^, for nude mice) or Lewis (2 × 10^5^, for C57BL mice) cells in 100-μl PBS intratracheally, as previously described [15]. The animals were anesthetized by intraperitoneal injection of pentobarbital sodium (50 mg/kg) prior to intratracheal injections (n = 5 in each group). For the FN treated mice model, we implanted FN pre-treated A549 (100 ng/ml, 72 h) tumor cells into nude mice, following with intratracheally instilling FN (100 ng in 100 μl PBS) to lung tissues on day 7 and day 14. In combination treatment, mice were treated with PBS, PTX (5 mg/kg) [16], MTX (2 mg/kg) [17], PTX (5 mg/kg) or MTX (2 mg/kg) combined with CT (2.5 mg/kg) by tail vein injection on days 7 and 14. According to previous reports, we chose a low reported doses for mice treatment to avoid potential toxicity in combination group. On days 30, 10 mice were sacrificed for the tumor weight analysis. The tumor weight was calculated as: tumor weight = total weight of lung - normal lung weight. The survival analysis was conducted by observing the tumor bearing mice every day until day 80. All our animal experiments were conducted in accordance with guidelines of Animal Ethics Committee and approved by the Institute Ethics Committee of Shanghai Ninth People's Hospital.

### 2.11 Statistical analysis

GraphPad Prism 6.0 was used for statistical analysis. Student's t-test and one-way ANOVA followed by the post hoc Tukey multiple comparisons test was used to analyze the between-group difference. In addition, the Kaplan–Meier method and the log-rank test were used for survival analysis. Notably, between-group difference with a two-sided P < 0.05 were considered significantly different.

## 3 Results

### 3.1 FN facilitates tumor growth in NSCLC

To investigate the role of FN in NSCLC progression, we obtained NSCLC tumors from patients with stage T0 and T3 NSCLC. Elevated expression of FN was observed in the tumor sites of high malignant patients (stage T3) compared with low malignant patients (stage T0) (Fig. 1A). This pattern was observed in multiple patients as evidenced by increased expression of FN in high malignant NSCLC patients (Fig. 1B; n = 10), indicating a potential association between FN and NSCLC progression. To further elucidate the pro-tumor effects induced by FN, we cultured NSCLC cells with either FN or PBS to investigate the effects of FN on NSCLC cell growth. Consistently, FN treatment significantly increased the proliferation of A549 and Lewis cells (Fig. 1C and D), both *in vitro* and *in vivo*. To further examine the tumorigenic potential of those NSCLC cells, we used a soft 3D fibrin gel to assess the colony formation capability [14]. Moreover, A549 and Lewis cells demonstrated enhanced colony-formation ability in the FN-treated group compared with the PBS group (Fig. 1E). Similarly, increased tumorigenesis capabilities in FN-treated NSCLC cells were observed *in vivo* (Fig. 1F), suggesting that FN could regulate NSCLC progression, leading to increased cell proliferation and tumorigenesis. Previous reports indicated that various cell types could produce FN, resulting in the remodeling of the microenvironment to regulate tumor physiological activities. Herein, we isolated different cell subpopulations, including cancer cells, immune cells and fibroblasts, from NSCLC tissues as described in the methods. Subsequently, FN expression was examined in these cell subpopulations. Intriguingly, elevated expression of FN was observed in CAFs, whereas cancer and immune cells produced low levels of FN (Fig. 1G). Additionally, we detected a significantly increased number of CAFs in high malignant tumor tissues compared to low malignant tumor tissues, suggesting that enriched CAFs in NSCLC tissues produced FN to promote tumor progression (Fig. 1H). Overall, these results indicated that FN could promote NSCLC cell proliferation, resulting in poor prognosis in patients.

**Fig. 1:**
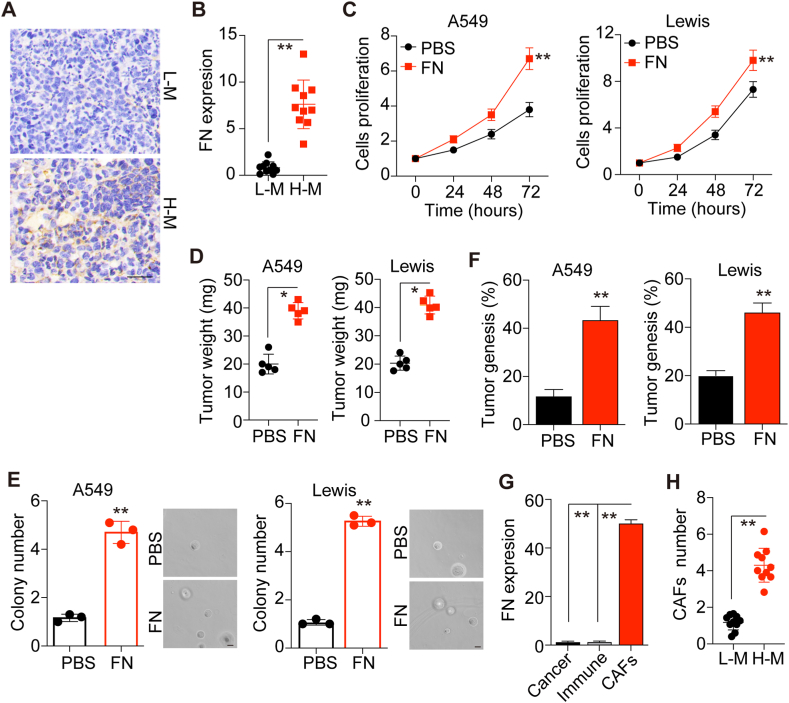
**FN promoted tumor growth in NSCLC. (**A) Immunohistochemistry of FN in high malignant (H–M) and low malignant (L–M) NSCLC patient tumor tissues. Scale bar, 100 μm. (B) Relative FN expression levels in NSCLC tumor tissues with H-M and L-M (n = 10). (C) Relative cell proliferation of A549 and Lewis cells treated with PBS or FN for 72 h. (D) The NSCLC tumor weight of mice. A total of 1 × 10^6^ A549 and Lewis cells pretreated with PBS or FN for 72 h were intratracheally instilled into nude mice. Tumor weight was calculated on day 20 and day 15 respectively. (E) Relative colony numbers and presentative images of A549 cells and Lewis cells treated with PBS or FN for 72 h. The scale bar is 30 μm. (F) The tumorigenesis of nude mice intratracheally instilled with 1 × 10^5^ A549 and Lewis cells pretreated with PBS or FN. (G) Relative FN expression in cancer cells, immune cells or CAFs isolated from tumor tissues of patients with NSCLC detected by qPCR. A549 cells were used as the internal control and normalized to the FN levels. (H) Relative CAF numbers in tumor tissues from NSCLC patients with H-M and L-M. Error bars, mean ± SEM; *P < 0.05; **P < 0.01; ns, not statistically significant.

### 3.2 FN promotes tumor growth via the activation of the integrin αvβ3/PI3K/AKT/SOX2 signaling pathway

Preliminary findings revealed that integrins, classical transmembrane glycoproteins that function as major receptors connecting the intracellular and extracellular environments, are highly correlated with pro-survival signal activation in tumor cells [10,11]. To investigate the potential role of integrin involved in FN-induced tumor progression, we examined the gene expression levels of integrin α5, α8, αv, and αII, which have been reported to bind to RGD-associated ECM [[18], [19], [20], [21]]. Significantly increased integrin αv expression was observed in FN-treated A549 and Lewis cells (Fig. 2A). Subsequently, we detected the gene expression levels of integrin β3, β5, β6, and β8, which can form heterodimers with integrin αv. Increased expression levels of β3 was observed in FN-treated A549 and Lewis cells (Fig. 2B). Meanwhile, we also observed the upregulation of integrin αvβ3 at the protein level in the FN treated-group by western blotting (Fig. 2C), suggesting that integrin αvβ3 participated in FN-induced tumor cell proliferation. To further demonstrate the role of integrin αvβ3, we treated tumor cells with an integrin avβ3 inhibitor, CT, and assess the FN-induced proliferation. CT significantly reversed the effects of FN in promoting proliferation and colony formation in A549 and Lewis cells (Fig. 2D, F). Consistently, similar results were observed *in vivo* (Fig. 2E, G). Additionally, we examined the αvβ3 expression in the tumor tissues of patients with NSCLC by immunofluorescence. Significantly increased integrin αvβ3 expression was observed in high malignant NSCLC tissues compared to low malignant NSCLC tissues (Fig. 2H). Altogether, these results suggested that FN could promote tumor growth via integrin αVβ3.

**Fig. 2:**
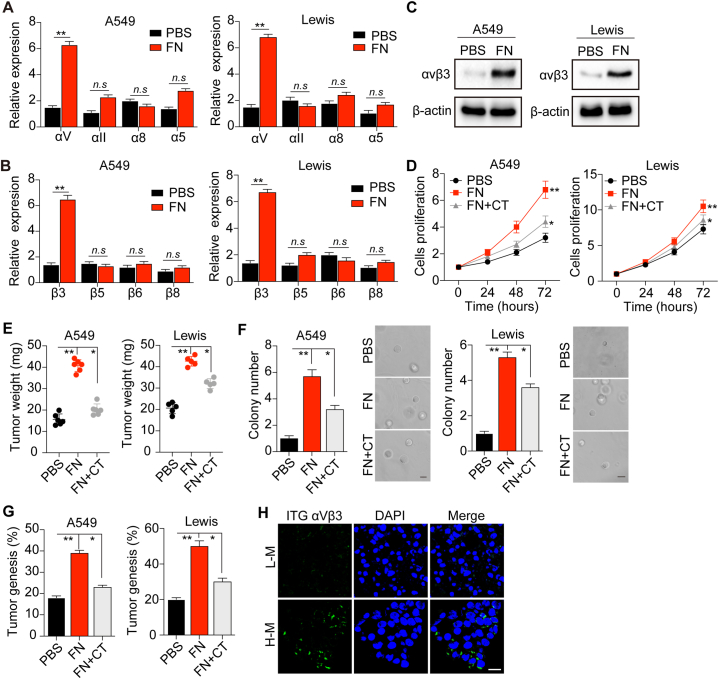
**FN promotes tumor growth through integrin αvβ3. (**A) The relative expression of integrin αv, αII, α8 and α5 in FN-treated or untreated A549 cells and Lewis cells for 72 h at the mRNA level. (B) The relative gene expression of integrin β3, β5, β6 and β8 in FN-treated or untreated A549 and Lewis cells for 72 h. (C) Western blot of integrin αvβ3 in A549 and Lewis cells treated or untreated with FN for 72 h. (D) Relative cell proliferation of A549 and Lewis cells treated with PBS, FN or FN plus CT for 72 h. (E) The NSCLC tumor weight of mice. A total of 1 × 10^6^ A549 and Lewis cells pretreated with PBS or FN or FN plus CT for 72 h were intratracheally instilled into nude mice. Tumor weight was calculated on day 20 and day 15 respectively. (F) Relative colony numbers and representative images of A549 and Lewis cells treated with PBS, FN or FN plus CT for 72 h. The scale bar is 30 μm. (G) The tumorigenesis of nude mice intratracheally instilled with 1 × 10^5^ A549 cells and Lewis cells pretreated with PBS, FN or FN plus CT for 72 h. (H) Immunofluorescence of integrin αvβ3 in tumor tissues of high malignant or low malignant NSCLC patients. Scale bar, 20 μm. Error bar, mean ± SEM; *P < 0.05; **P < 0.01; ns, not statistically significant. Cilengitide, CT.

### 3.3 FN facilitates tumor progression via the PI3K/AKT signaling pathway

As a classical downstream signaling pathway of integrins, the PI3K/AKT pathway plays a vital role in various physiological activities, including cell proliferation, survival and migration [22]. Previous studies showed that the PI3K/AKT signaling pathway is closely associated with the sustained growth of a variety of solid tumors [23]. Consistently, we observed increased phosphorylated PI3K and AKT in FN-treated A549 and Lewis cells at the protein level by western blotting (Fig. 3A), suggesting the activation of the PI3K/AKT signaling pathway in NSCLC cells treated with FN. To further verify the role of the PI3K/AKT signaling pathway, we used the PI3K inhibitor MK2206 and AKT inhibitor LY294002 to inhibit PI3K/AKT signaling. As expected, inhibition of the PI3K/AKT signaling pathway significantly reversed the enhanced proliferation and colony formation abilities induced by FN (Fig. 3B and C). In addition, similar results were observed *in vivo* (Fig. 3D and E). Consistently, increased p-PI3K and p-AKT levels were observed in high malignant NSCLC tumor tissues (Fig. 3F). These findings demonstrated that integrin αvβ3 induced tumor progression via the PI3K/AKT signaling pathway in NSCLC.

**Fig. 3:**
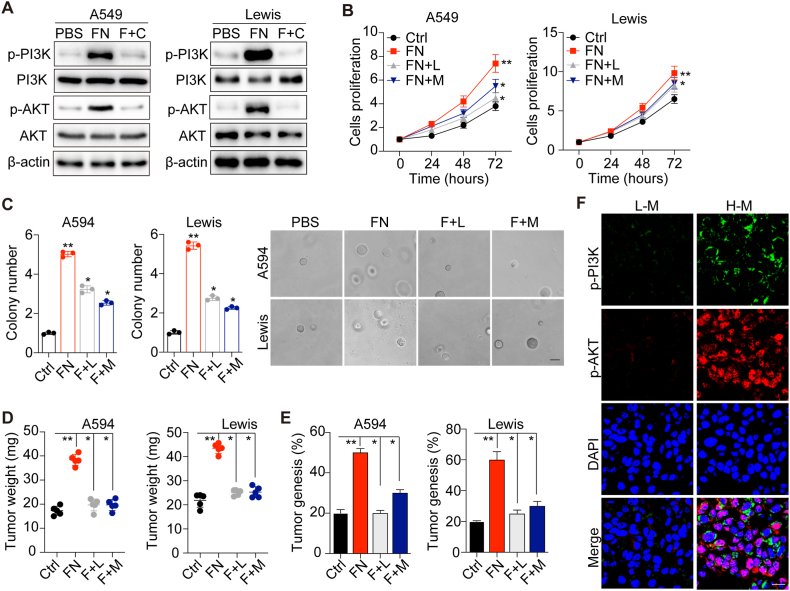
**FN facilitates tumor progression via the PI3K/AKT signaling pathway. (**A) Western blotting of p-PI3K, total-PI3K, p-AKT, total AKT and actin in A549 and Lewis cells treated with PBS, FN, FN plus CT for 72 h. (B) The relative cell proliferation of A549 and Lewis cells treated with PBS, FN, FN combined with LY204002 or MK2206 for 72 h. C, the relative colony numbers and representative images of A549 and Lewis cells treated with PBS, FN, FN combined with LY204002 or MK2206 for 72 h. The scale bar is 30 μm. D, the NSCLC tumor weight of mice, of note, 10^6^ A549 and Lewis cells pretreated with PBS, FN, FN combined with LY204002 or MK2206 for 72 h were intratracheally instilled into nude mice. The tumor weight was calculated on day 20 and day 15 respectively. E, the tumorigenesis of nude mice on day 30, of note, 10^5^ A549 and Lewis cells pretreated with PBS, FN, FN combined with LY204002 or MK2206 for 72 h were intratracheally instilled into nude mice. F, the immunofluorescence of p-PI3K and p-AKT in tumor tissues from NSCLC patients in stage T0 or stage T3. Scale bar, 20 μm. Error bars, mean ± SEM; *P < 0.05; **P < 0.01; ns, not statistically significant. LY204002, L. MK2206, M.

### 3.4 FN-induced tumor progression by the activation of PI3K/AKT/SOX2 signal pathway

Tumor cell pro-survival typically results in the upregulation of stem cell transcription factors, such as SOX2, Oct3/4, c-Myc and Nanog [24]. Moreover, AKT-driven phosphorylation was reported to maintain the stability and nuclear localization of SOX2 protein, which is functionally associates with enhanced cell proliferation and anti-apoptosis, thereby regulating clonogenicity and tumorigenicity in cancer [25]. Furthermore, SOX2 has been demonstrated to act as a molecular prognostic factor in NSCLC [26]. To assess whether the upregulation of stem cell transcription factors participate in integrin αvβ3/PI3K/AKT-induced tumor cell proliferation, we measured the gene expression levels of SOX2, Oct3/4, c-Myc, Tert, KLF4 and Nanog in PBS- or FN-treated A549 and Lewis cells by real-time PCR. Significantly increased gene expression level of SOX2 was observed in FN-pretreated A549 and Lewis cells compared with the PBS-treated group (Fig. 4A). Meanwhile, we found that the expression of KLF4 was upregulated. Herein, we performed immunofluorescence staining of KLF4 in tumor tissues from patients. However, no significant difference was observed in those tumor tissues from patients (Fig. S1A). We speculated that tumor cells in those tumor tissues might regulated the tumor stemness through other transcription factors rather than KLF4. Next, we focus on the role of SOX2 in tumor development. Similar results of real-time PCR were observed in immunofluorescence staining assays (Fig. 4B). Therefore, we hypothesized that FN facilitates tumor growth through SOX2, as the downstream transcription factor of PI3K/AKT in NSCLC. To further verify this, we silenced SOX2 gene expression in A549 and Lewis cells using siRNA (Fig. S1B). SOX2 silencing significantly reversed FN-induced cell proliferation and colony-formation abilities (Fig. 4C and D) compared with the scramble group. Similar results were observed *in vivo* (Fig. 4E and F). To further investigate the potential clinical value, we examined the SOX2 expression levels in tumor tissues of NSCLC patients by immunofluorescence staining. Significantly increased SOX2 expression was observed in high malignant tumor tissue compared with low malignant tissues (Fig. 4G). Taken together, our findings suggested that FN might activate the integrin αvβ3/PI3K/AKT/SOX2 signaling pathway to facilitate the proliferation of NSCLC cells.

**Fig. 4:**
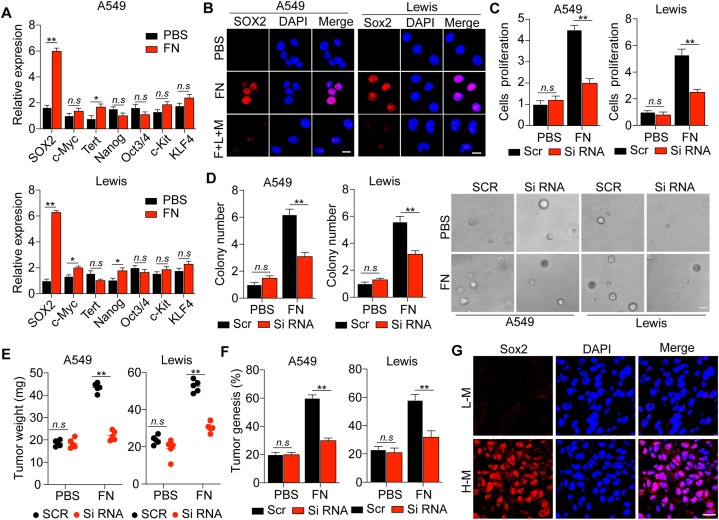
**FN-induced tumor progression by the activation of PI3K/AKT/SOX2 signal pathway.** A, The relative gene expression of SOX2, c-Myc, Tert, Nanog, Oct3/4 and KLF4 in A549 and Lewis cells treated with PBS or FN for 72 h. (B) Immunofluorescence of SOX2 in A549 and Lewis cells treated with PBS, FN or FN plus LY204002 and MK2206 for 72 h. Scale bar, 15 μm. (C) The relative cell proliferation of scramble (Scr) and SOX2-siRNA-A549 and Lewis cells treated with PBS or FN for 72 h. (D) The relative colony numbers and representative images of scramble (Scr) and SOX2-siRNA-A549 and Lewis cells treated with PBS or FN for 72 h. The scale bar is 30 μm. (E) The tumor weight of NSCLC-bearing mice. A total of 1 × 10^6^ scramble (Scr) or SOX2-siRNA-A549 cells and scramble (Scr) or SOX2-siRNA-Lewis cells pretreated with PBS or FN for 72 h were intratracheally instilled into mice. The tumor weight was calculated on day 20 and day 15 respectively. (F) The tumorigenesis of mice intratracheally instilled with 1 × 10^5^ scramble (Scr) or SOX2-siRNA-A549 cells amd scramble (Scr) or SOX2-siRNA-Lewis cells pretreated with PBS or FN for 72 h. (G) The immunofluorescence of SOX2 in tumor tissues of NSCLC patients in stage T0 or T3. Scale bar, 20 μm. Error bars, mean ± SEM; *P < 0.05; **P < 0.01; ns, not statistically significant. LY204002, L. MK2206, M.

### 3.5 Combination of integrin αvβ3 inhibitor and chemotherapeutic agents revealed enhanced anticancer effects *in vivo*

Considering the role of the integrin αvβ3/PI3K/AKT/SOX2 signaling pathway in FN-induced proliferation, we hypothesized that the combination of integrin αvβ3 inhibitor and chemotherapeutic agents might serve as a potential clinical therapeutic strategy in NSCLC. To verify this hypothesis, we established an NSCLC mouse model by intratracheally injecting 2 × 10^6^ A549 cells. Subsequently, we treated mice with PBS, PTX or PTX combined with CT by intratracheal instillation. Significant tumor suppression effects were observed in the PTX combined with CT group compared with the PBS or PTX groups (Fig. 5A). Furthermore, mice treated with CT and PTX revealed significantly prolonged survival time compared with the PBS or PTX groups (Fig. 5B). Similar enhanced tumor suppression effects were also observed in MTX combined with CT treatment groups (Fig. 5C and D) and Lewis-bearing mice models (Fig. 5E and F). To further investigate the potential clinical value of integrin αvβ3 inhibitor-based combination therapy in relatively high malignant patients with NSCLC, we established an FN-treated NSCLC mouse model by instilling 2 × 10^6^ FN-pretreated A549 cells through intratracheal instillation. We then treated mice with PBS, PTX or PTX combined with CT by intratracheal instillation. Significantly enhanced tumor suppression effects and prolonged survival time were observed in the combination treatment group compared with the PBS or PTX groups, whereas single chemotherapy revealed limited anticancer effects (Fig. 5G and H). Taken together, these findings suggested that integrin αvβ3 inhibitor improved the outcome of chemotherapeutic agents in NSCLC.

**Fig. 5:**
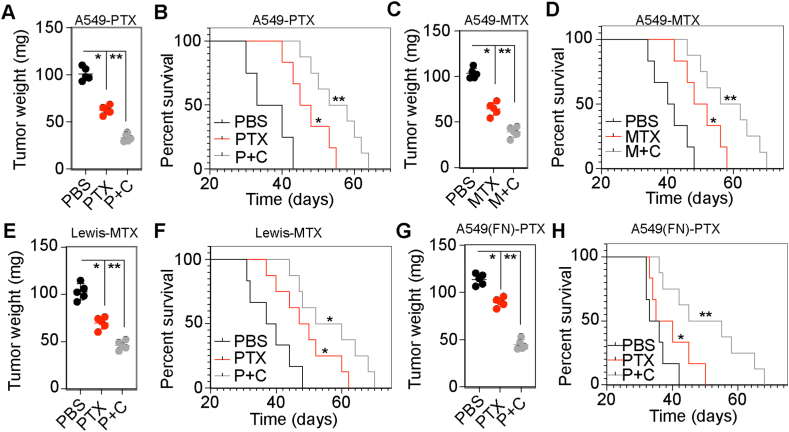
**Combination of integrin αvβ3 inhibitor and chemotherapeutic agents enhances anticancer effects in NSCLC treatment. (**A) The tumor weight analysis of lung tissues from mice on day 35 after intratracheally instillation withA549 cells and treatment with PBS, PTX or PTX combined with CT. (B) The survival time of A549-bearing mice treated with PBS, PTX or PTX combined with CT. (C) The NSCLC tumor weight of mice on day 35 after intratracheally instillation with A549 cells and treatment with PBS, MTX or MTX combined with CT. (D) The survival time of A549-bearing mice treated with PBS, MTX or MTX combined with CT. (E) The NSCLC tumor weight of mice on day 25 after intratracheally instillation with Lewis cells and treatment with PBS, PTX or PTX combined with CT. (F) The survival time of Lewis-bearing mice treated with PBS, PTX or PTX combined with CT. (G) The FN-treated NSCLC tumor weight of mice on day 35 after intratracheally instillation with 1 × 10^6^ A549 cells and treatment with PBS, PTX or PTX combined with CT. (H) The survival time of FN-treated A549 bearing mice treated with PBS, PTX or PTX combined with CT. Error bars, mean ± SEM; *P < 0.05; **P < 0.01; ns, not statistically significant. Cilengitide, CT.

## 4 Discussion

FN has emerged as a significant extracellular driver of malignancy in recent years. Previous studies reported that FN plays a crucial role in various malignant behaviors in solid tumors, including tumor invasion and cancer cell migration and proliferation [12,27]. Although the integrin β3 receptor has been reported to play a key role in the binding and activation of intercellular signaling pathways, the molecular mechanism underlying FN-facilitating tumor cell proliferation remains poorly characterized [11,28]. Recent studies suggested that FN could interact with integrin α5β1 to activate focal adhesion kinase (FAK), resulting in a downstream invasion cascade [29]. However, the molecular mechanism underlying FN-facilitating tumor cell proliferation remains poorly characterized, even though the integrin β3 receptor has been reported to play a key role in the binding and activation of intercellular signaling pathways [30].

The significant finding in this study lies in the identification that FN promoted tumor growth through its interaction with integrin αvβ3, further activating the downstream PI3K/AKT signaling pathway and upregulating SOX2 expression, eventually leading to the sustained proliferation and enhanced tumorigenesis in cancer cells. Previous reports revealed that several cytokines, such as IGF2, could facilitate NSCLC stemness via the PI3K/AKT signaling pathway [31,32]. Additionally, AKT signaling is markedly associated with the development of drug resistance in various tumor types, including lung cancer [33,34]. We associated FN with the PI3K/AKT signaling pathway, which further revived the underlying mechanism of ECM-induced tumor development. Accordingly, an intriguing question arising from these discoveries is the cellular source of FN in the tumor microenvironment. Previous reports hypothesized that FN largely originated from the tumor cells themselves, since many tumors could produce FN when samples were cultured *ex vivo* [35]. However, there are various types of cells in the tumor microenvironment, including immune cells, endothelial cells and fibroblasts [36]. In this study, we verified that FN was mainly produced by CAFs, instead of tumor or immune cells in tumor tissues. Fundamental understanding of FN-promoted tumor growth and the underlying molecular pathway can lead to the development of molecular targeted combination therapy in patients with NSCLC. More importantly, previous studies demonstrated that traditional chemotherapy or single CT treatment failed in tumor suppression treatment. In this study, we provided evidence that the combination of the integrin αvβ3 inhibitor CT and chemotherapeutic agents showed significant tumor suppressive effects in NSCLC treatment, which suggested a potential combination therapeutic strategy for patients with NSCLC and the clinical application of integrin αvβ3 inhibitors.

Based on the limitations of previous reports, we aimed to provide a more comprehensive understanding of the role of FN in NSCLC development. Firstly, our study disclosed the association between FN and sustained tumor growth, demonstrating that elevated FN expression by CAFs could result in malignant NSCLC development. Secondly, we explored the underlying mechanism of tumor progression induced by FN. It was showed that FN promoted tumor cell proliferation through activation of the PI3K/AKT/SOX2 signaling pathway in NSCLC. Thirdly, it was further identified that the combination of CT and chemotherapeutic agents could significantly improve the outcome in NSCLC treatment. Compared to single CT or chemotherapeutic agents, the combination strategy is more suitable in clinical NSCLC treatment and provides an innovative approach. Finally, FN expression levels in NSCLC tissues might serve as a potential biomarker for clinical NSCLC progression analysis. However, non-cancer cells have not been examined for the expression of integrins or FN, which should be further analyzed before clinical biomarkers detection. And the role of other extracellular matrix proteins in the progression of NSCLC, such as type V collagen, should be further investigated. Additionally, the combination of CT and chemotherapy should be further validated in further clinical trials.

In summary, this study elucidated the role of FN in the NSCLC development. FN could regulate NSCLC progression through the integrin αvβ3/PI3K/AKT/SOX2 signaling pathway. Inhibition of integrin αvβ3 significantly suppressed NSCLC growth, which might serve as a novel therapeutic strategy in the NSCLC treatment.

## Ethics statement

The study was approved by the Ethics Committee of Shanghai Ninth People's Hospital.

## Author contribution statement

Jin-Long Wu: Conceived and designed the experiments; Performed the experiments; Analyzed and interpreted the data; Contributed reagents, materials, analysis tools or data; Wrote the paper. Ming-Song Wang: Conceived and designed the experiments; Wrote the paper. Cheng-Feng Xu: Performed the experiments; Analyzed and interpreted the data; Contributed regents, materials, analysis tools or data. Xu-Hui Yang: Contributed reagents, materials, analysis tools or data.

## Data availability statement

Data included in article/supp. Material/referenced in article.

## Funding

This study was supported by the 10.13039/501100001809National Natural Science Foundation of China (82072567).

## Declaration of competing interest

The authors declare that they have no known competing financial interests or personal relationships that could have appeared to influence the work reported in this paper.

## Figures and Tables

**figs1:**
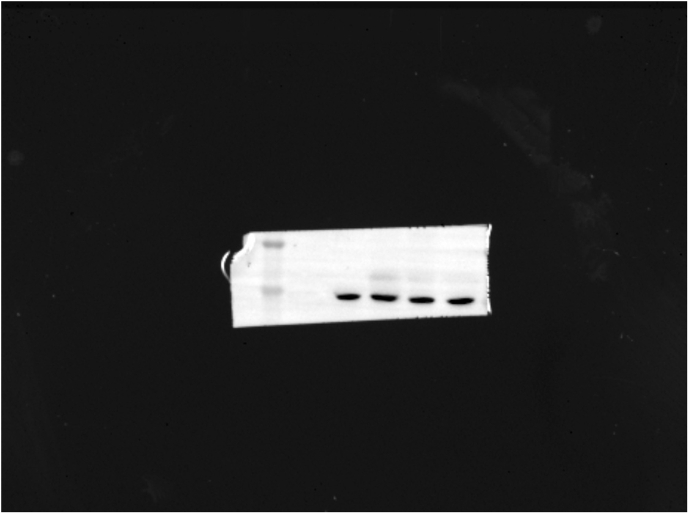


**figs2:**
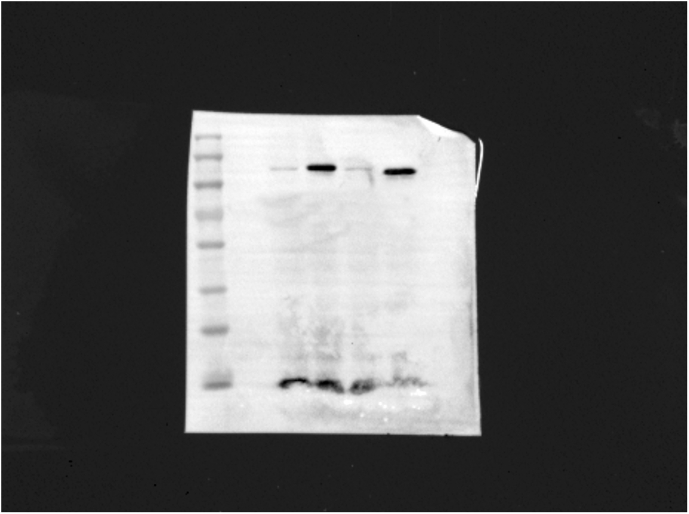


**figs3:**
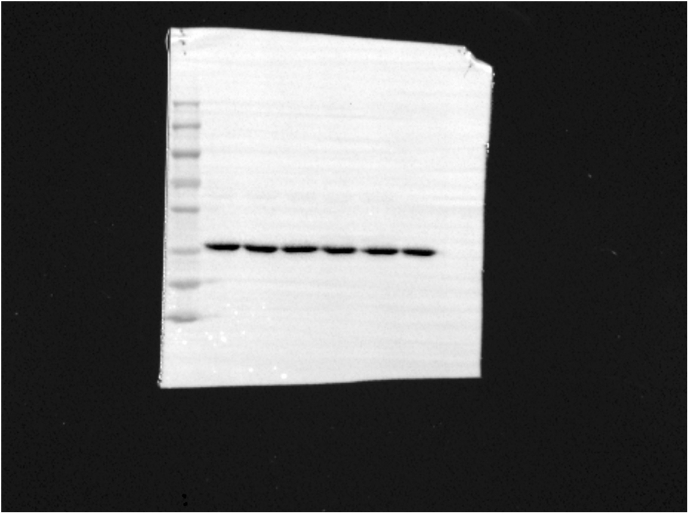


**figs4:**
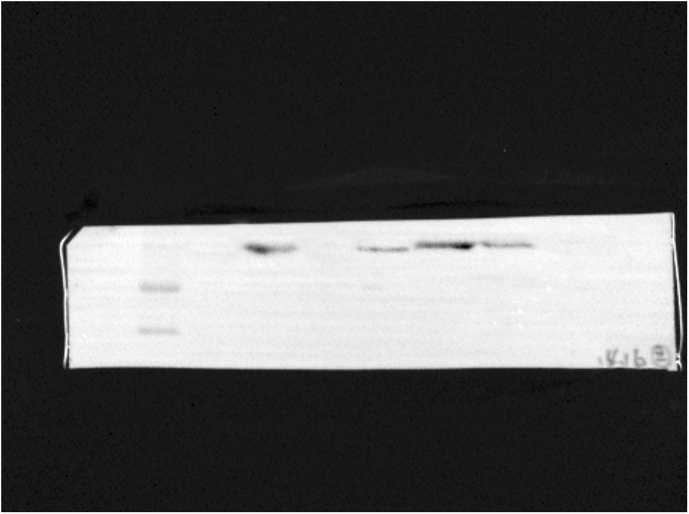


**figs5:**
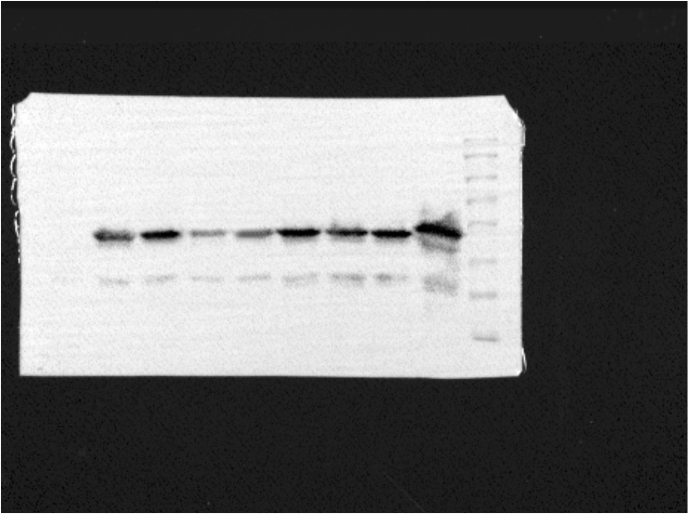


**figs6:**
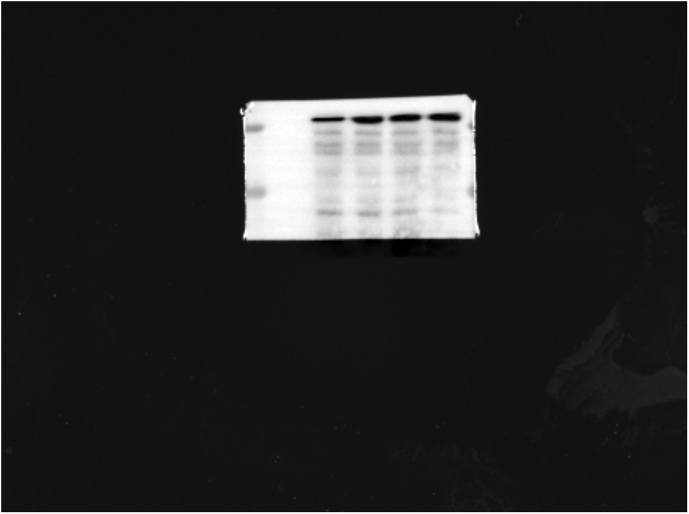


**figs7:**
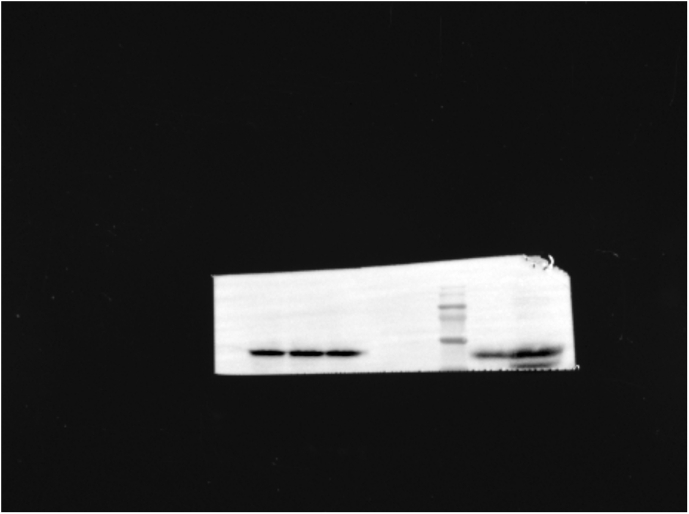


**figs8:**
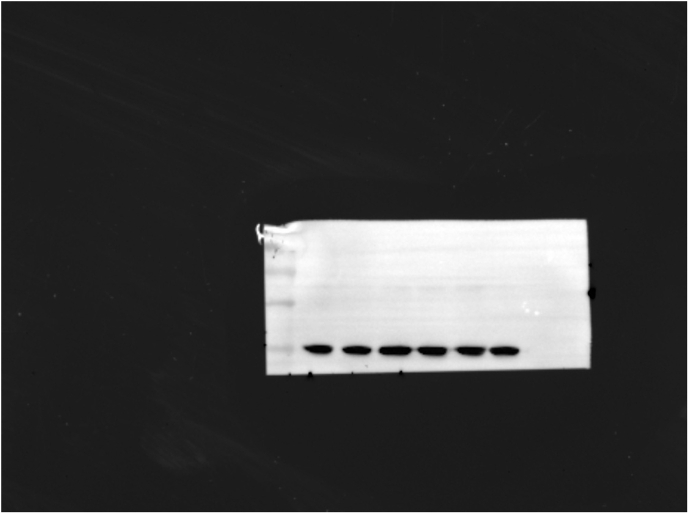


**figs9:**
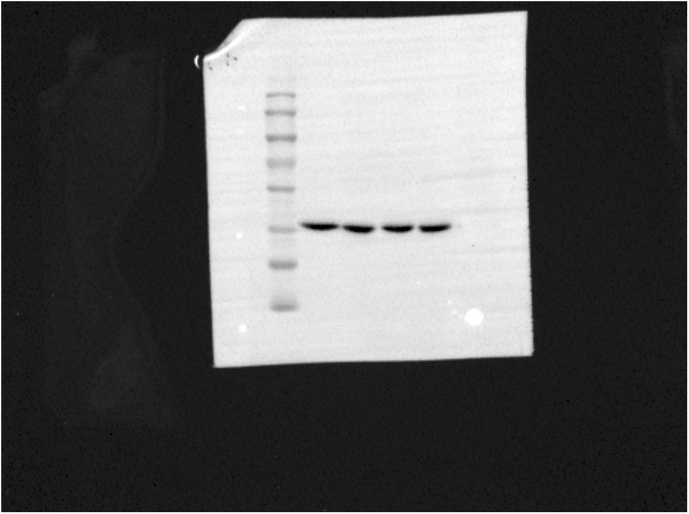


**figs10:**
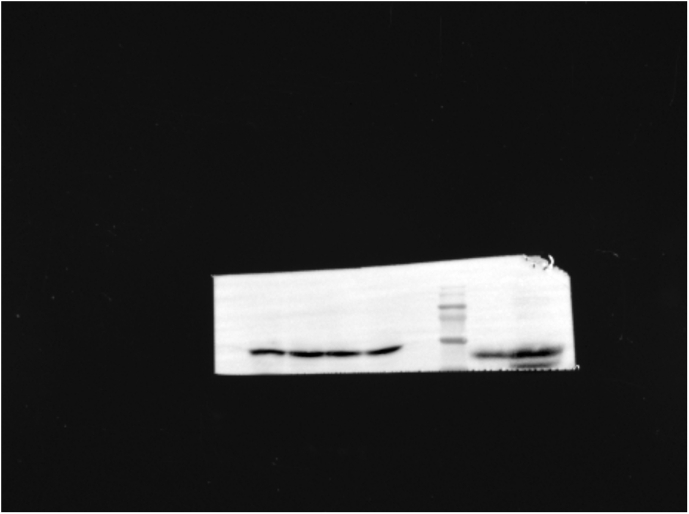


**figs11:**
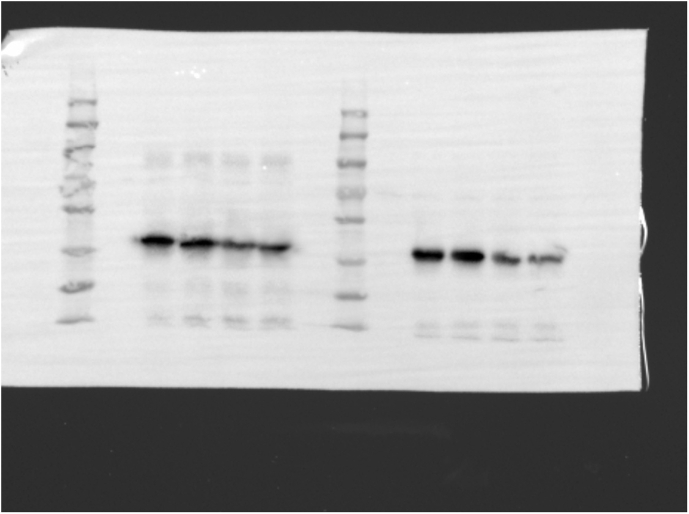


## References

[1] Torre L.A., Siegel R.L., Jemal A., Lung Cancer Statistics (2016). Adv. Exp. Med. Biol..

[2] Travis W.D. (2002). Pathology of lung cancer. Clin. Chest Med..

[3] Nagasaka M., Gadgeel S.M. (2018). Role of chemotherapy and targeted therapy in early-stage non-small cell lung cancer. Expet Rev. Anticancer Ther..

[4] Fry W.A., Phillips J.L., Menck H.R. (1999). Ten-year survey of lung cancer treatment and survival in hospitals in the United States: a national cancer data base report. Cancer.

[5] Strauss G.M. (2005). Adjuvant chemotherapy of lung cancer: methodologic issues and therapeutic advances. Hematol. Oncol. Clin. N. Am..

[6] Chang A. (2011). Chemotherapy, chemoresistance and the changing treatment landscape for NSCLC. Lung Cancer.

[7] Whiteside T.L. (2008). The tumor microenvironment and its role in promoting tumor growth. Oncogene.

[8] Buchheit C.L., Weigel K.J., Schafer Z.T. (2014). Cancer cell survival during detachment from the ECM: multiple barriers to tumour progression. Nat. Rev. Cancer.

[9] Kim S.H., Turnbull J., Guimond S. (2011). Extracellular matrix and cell signalling: the dynamic cooperation of integrin, proteoglycan and growth factor receptor. J. Endocrinol..

[10] Miyamoto S., Teramoto H., Coso O.A., Gutkind J.S., Burbelo P.D., Akiyama S.K., Yamada K.M. (1995). Integrin function: molecular hierarchies of cytoskeletal and signaling molecules. J. Cell Biol..

[11] Rathinam R., Alahari S.K. (2010). Important role of integrins in the cancer biology. Cancer Metastasis Rev..

[12] Gopal S., Veracini L., Grall D., Butori C., Schaub S., Audebert S., Camoin L., Baudelet E., Radwanska A., Beghelli-de la Forest Divonne S. (2017). Fibronectin-guided migration of carcinoma collectives. Nat. Commun..

[13] Mitra A.K., Sawada K., Tiwari P., Mui K., Gwin K., Lengyel E. (2011). Ligand-independent activation of c-Met by fibronectin and alpha(5)beta(1)-integrin regulates ovarian cancer invasion and metastasis. Oncogene.

[14] Liu J., Tan Y., Zhang H., Zhang Y., Xu P., Chen J., Poh Y.C., Tang K., Wang N., Huang B. (2012). Soft fibrin gels promote selection and growth of tumorigenic cells. Nat. Mater..

[15] Li Y., Cao F., Li M., Li P., Yu Y., Xiang L., Xu T., Lei J., Tai Y.Y., Zhu J. (2018). Hydroxychloroquine induced lung cancer suppression by enhancing chemo-sensitization and promoting the transition of M2-TAMs to M1-like macrophages. J. Exp. Clin. Cancer Res..

[16] Zhang X., Zhang R., Huang J., Luo M., Chen X., Kang Y., Wu J. (2019). Albumin enhances PTX delivery ability of dextran NPs and therapeutic efficacy of PTX for colorectal cancer. J. Mater. Chem. B.

[17] Choudhury R.C., Ghosh S.K., Palo A.K. (2000). Cytogenetic toxicity of methotrexate in mouse bone marrow. Environ. Toxicol. Pharmacol..

[18] Diamond M.S., Springer T.A. (1994). The dynamic regulation of integrin adhesiveness. Curr. Biol..

[19] Humphries J.D., Byron A., Humphries M.J. (2006). Integrin ligands at a glance. J. Cell Sci..

[20] Gahmberg C.G., Fagerholm S.C., Nurmi S.M., Chavakis T., Marchesan S., Grönholm M. (2009). Regulation of integrin activity and signalling. Biochim. Biophys. Acta Gen. Subj..

[21] Madamanchi A., Santoro S.A., Zutter M.M., Integrin α2β1 (2014). Adv. Exp. Med. Biol..

[22] Osaki M., Oshimura M., Ito H. (2004). PI3K-Akt pathway: its functions and alterations in human cancer. Apoptosis : an international journal on programmed cell death.

[23] Fresno Vara J.A., Casado E., de Castro J., Cejas P., Belda-Iniesta C., Gonzalez-Baron M. (2004). PI3K/Akt signalling pathway and cancer. Cancer Treat Rev..

[24] Liu A., Yu X., Liu S. (2013). Pluripotency transcription factors and cancer stem cells: small genes make a big difference. Chin. J. Cancer.

[25] Schaefer T., Lengerke C. (2019). SOX2 protein biochemistry in stemness, reprogramming, and cancer: the PI3K/AKT/SOX2 axis and beyond. Oncogene.

[26] Xiang R., Liao D., Cheng T., Zhou H., Shi Q., Chuang T.S., Markowitz D., Reisfeld R.A., Luo Y. (2011). Downregulation of transcription factor SOX2 in cancer stem cells suppresses growth and metastasis of lung cancer. Br. J. Cancer.

[27] Rick J.W., Chandra A., Dalle Ore C., Nguyen A.T., Yagnik G., Aghi M.K. (2019). Fibronectin in malignancy: cancer-specific alterations, protumoral effects, and therapeutic implications. Semin. Oncol..

[28] Paolillo M., Schinelli S. (2019). Extracellular matrix alterations in metastatic processes. Int. J. Mol. Sci..

[29] Mitra S.K., Schlaepfer D.D. (2006). Integrin-regulated FAK-Src signaling in normal and cancer cells. Curr. Opin. Cell Biol..

[30] Schwartz M.A. (2001). Integrin signaling revisited. Trends Cell Biol..

[31] Fernandez L.A., Squatrito M., Northcott P., Awan A., Holland E.C., Taylor M.D., Nahle Z., Kenney A.M. (2012). Oncogenic YAP promotes radioresistance and genomic instability in medulloblastoma through IGF2-mediated Akt activation. Oncogene.

[32] Navab R., Strumpf D., To C., Pasko E., Kim K.S., Park C.J., Hai J., Liu J., Jonkman J., Barczyk M. (2016). Integrin alpha11beta1 regulates cancer stromal stiffness and promotes tumorigenicity and metastasis in non-small cell lung cancer. Oncogene.

[33] He K., Xu T., Xu Y., Ring A., Kahn M., Goldkorn A. (2014). Cancer cells acquire a drug resistant, highly tumorigenic, cancer stem-like phenotype through modulation of the PI3K/Akt/beta-catenin/CBP pathway. Int. J. Cancer.

[34] Zhou G., Zhang F., Guo Y., Huang J., Xie Y., Yue S., Chen M., Jiang H., Li M. (2017). miR-200c enhances sensitivity of drug-resistant non-small cell lung cancer to gefitinib by suppression of PI3K/Akt signaling pathway and inhibites cell migration via targeting ZEB1. Biomedicine & pharmacotherapy = Biomedecine & pharmacotherapie.

[35] Ruoslahti E. (1984). Fibronectin in cell adhesion and invasion. Cancer Metastasis Rev..

[36] To W.S., Midwood K.S. (2011). Plasma and cellular fibronectin: distinct and independent functions during tissue repair. Fibrogenesis Tissue Repair.

