# Supplementary material for: Fibronectin promotes tumor progression through integrin αvβ3/PI3K/AKT/SOX2 signaling in non-small cell lung cancer
Source: Heliyon. 2023 Sep 14;9(9):e20185. doi: 10.1016/j.heliyon.2023.e20185 (PMC10559956; doi:10.1016/j.heliyon.2023.e20185)
Supplement: Multimedia component 1 [file mmc1.docx]

**Supplementary figure**

**
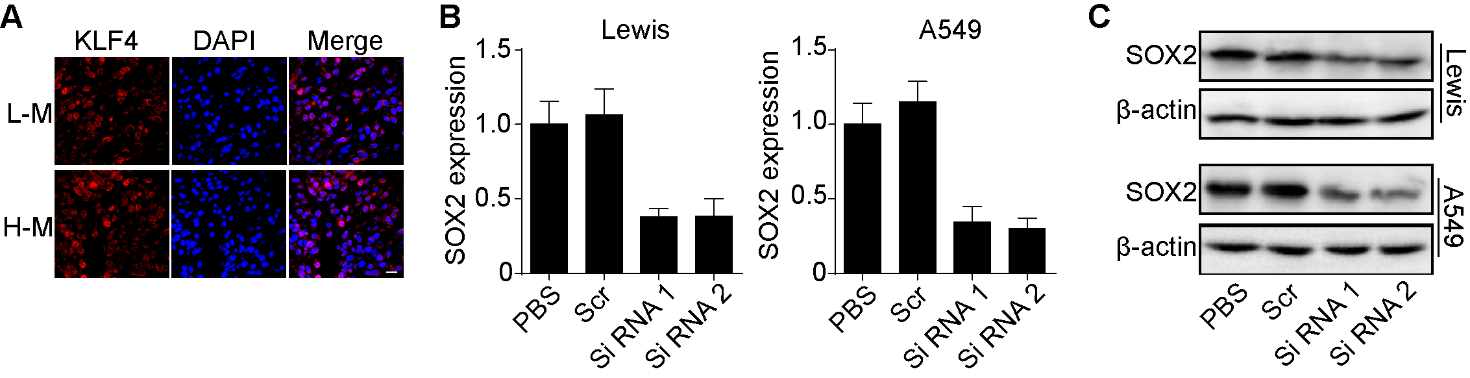
**

**Figure S1.** **FN induces tumor progression activating the PI3K/AKT/SOX2 signaling pathway.** (A) The immunofluorescence of KLF4 in high malignant (H-M) and low malignant (L-M) tumor tissues from patients. Scale bar, 15 μm. (B and C) The SOX2 expression at mRNA and protein level of Lewis and A549 cells treated with scramble (Scr) and SOX2-siRNA. Error bars, mean ± SEM; **P*<0.05; ***P*<0.01; ns, not statistically significant.

**Supplementary methods**

**Table 1**

Patient information

| **Variable** | | **Patients number (total n=20)** |
| --- | --- | --- |
| Age | >65 | 12 (60%) |
|  | <65 | 8 (40%) |
| Gender | Male | 18 (90%) |
|  | Female | 2 (10%) |
| Types | Adenocarcinoma | 20 (100%) |
| Stage | Stage I (L-M) | 10 (50%) |
|  | Stage III (H-M) | 10 (50%) |
| Distant metastasis | M0 | 20 (100%) |
|  | M1 | 0 (0%) |

**Table 2**

Primer sequence

| Gene name | Forward primer | Reverse primer |
| --- | --- | --- |
| human integrin α5 | 5’-GGCTTCAACTTAGACGCGGAG-3’ | 5’-TGGCTGGTATTAGCCTTGGGT-3’ |
| human integrin α8 | 5’-TCAGGCGTTCAACCTGGAC-3’ | ’-GCGTCGGGTATGTGGAAGTC-3’ |
| human integrin αV | 5’-ATCTGTGAGGTCGAAACAGGA-3’ | 5’-TGGAGCATACTCAACAGTCTTTG-3’ |
| human integrin αII | 5’-GTGGCAATAAGTGGCTGGTC-3’ | 5’-GTTCCCGTGGATCACTGGAC-3’ |
| human integrin β3 | 5’-GTGACCTGAAGGAGAATCTGC-3’ | 5’-CCGGAGTGCAATCCTCTGG-3’ |
| human integrin β5 | 5’-TCTCGGTGTGATCTGAGGG-3’ | 5’-TGGCGAACCTGTAGCTGGA-3’ |
| human integrin β6 | 5’-TCCATCTGGAGTTGGCGAAAG-3’ | 5’-TCTGTCTGCCTACACTGAGAG-3’ |
| human integrin β8 | 5’-ACCAGGAGAAGTGTCTATCCAG-3’ | 5’-CCAAGACGAAAGTCACGGGA-3’ |
| murine integrin α5 | 5’-TGCAGTGGTTCGGAGCAAC-3’ | 5’-TTTTCTGTGCGCCAGCTATAC-3’ |
| murine integrin α8 | 5’-TGTCTGGCGTTCAACTTGGAT-3’ | 5’-TCCAGTGAGTAGCCGAAGTAG-3’ |
| murine integrin αv | 5’-CGGGTCCCGAGGGAAGTTA-3’ | 5’-TGGATGAGCATTCACATTTGAGA-3' |
| murine integrin αII | 5’-TGCCCCAATGGAAACCAATG-3’ | 5’-CATGCCAGTGGTGTAGTAGGA-3’ |
| murine integrin β3 | 5’-GGCGTTGTTGTTGGAGAGTC-3’ | 5’-CTTCAGGTTACATCGGGGTGA-3' |
| integrin β5 | 5’-GAAGTGCCACCTCGTGTGAA-3’ | 5’-GGACCGTGGATTGCCAAAGT-3' |
| murine integrin β6 | 5’-ATGGGGATTGAGCTGGTCTG-3’ | 5’-GACAGGTGGGTGAAATTCTCC-3' |
| murine integrin β8 | 5’-TGCATGTTGTAACGTCAAGTGA-3’ | 5’-GATGCTGACACATCAACCAGATA-3' |
| human SOX2 | 5’-GCCGAGTGGAAACTTTTGTCG-3’ | 5’-GGCAGCGTGTACTTATCCTTCT-3’ |
| human c-Myc | 5’-GGCTCCTGGCAAAAGGTCA-3’ | 5’-CTGCGTAGTTGTGCTGATGT-3' |
| human Tert | 5’-AAATGCGGCCCCTGTTTCT-3’ | 5’- CAGTGCGTCTTGAGGAGCA-3’ |
| human Nanog | 5’-TTTGTGGGCCTGAAGAAAACT-3’ | 5’- AGGGCTGTCCTGAATAAGCAG-3’ |
| human Oct3/4 | 5’-CTGGGTTGATCCTCGGACCT-3’ | 5’-CCATCGGAGTTGCTCTCCA-3’ |
| murine Sox2 | 5’-GCGGAGTGGAAACTTTTGTCC-3’ | 5’-GGGAAGCGTGTACTTATCCTTCT-3' |
| murine c-Myc | 5’-ATGCCCCTCAACGTGAACTTC-3’ | 5’- GTCGCAGATGAAATAGGGCTG-3’ |
| murine Tert | 5’-TCTACCGCACTTTGGTTGCC-3’ | 5- CAGCACGTTTCTCTCGTTGC-3’ |
| murine Nanog | 5’-CACAGTTTGCCTAGTTCTGAGG-3’ | 5’-GCAAGAATAGTTCTCGGGATGAA-3’ |
| murine Oct-4 | 5’-AGAGGATCACCTTGGGGTACA-3’ | 5’-CGAAGCGACAGATGGTGGTC-3' |
| human KLF4 | 5’-CCCACATGAAGCGACTTCCC-3’ | 5’-CAGGTCCAGGAGATCGTTGAA-3’ |
| murine KLF4 | 5’-GGCGAGTCTGACATGGCTG-3’ | 5’-GCTGGACGCAGTGTCTTCTC-3’ |
| human GAPDH | 5'-GGAGCGAGATCCCTCCAAAAT-3' | 5'-GGCTGTTGTCATACTTCTCATGG-3' |
| murine GAPDH | 5'- GAPDHAGGTCGGTGTGAACGGATTTG-3' | 5’-GGGGTCGTTGATGGCAACA-3' |
